# Describing the dynamic translational science landscape through Core Voucher utilization

**DOI:** 10.1017/cts.2019.4

**Published:** 2019-06-14

**Authors:** Elvira L. Liclican, Scott G. Filler, Jonathan Kaye, Christopher T. Denny

**Affiliations:** 1 UCLA Clinical and Translational Science Institute, University of California, Los Angeles, Los Angeles, CA, USA; 2 Division of Infectious Diseases, Los Angeles Biomedical Research Institute at Harbor-UCLA Medical Center, Torrance, CA, USA; 3 Research Division of Immunology, Departments of Biomedical Sciences and Medicine, Samuel Oschin Comprehensive Cancer Institute, Cedars-Sinai Medical Center, Los Angeles, CA, USA; 4 Department of Medicine, David Geffen School of Medicine, University of California, Los Angeles, CA, USA; 5 Division of Hematology/Oncology, Department of Pediatrics, Gwynne Hazen Cherry Memorial Laboratories, University of California, Los Angeles, CA, USA; 6 Molecular Biology Institute, University of California, Los Angeles, CA, USA; Jonsson Comprehensive Cancer Center, University of California, Los Angeles, CA, USA; 7 California NanoSystems Institute, University of California, Los Angeles, CA, USA

**Keywords:** Core facility, Core Voucher, translational science, UCLA CTSI, request for application

## Abstract

**Introduction:**

Core facilities play crucial roles in carrying out the academic research mission by making available to researchers advanced technologies, facilities, or expertise that are unfeasible for most investigators to obtain on their own. To facilitate translational science through support of core services, the University of California, Los Angeles Clinical and Translational Science Institute (UCLA CTSI) created a Core Voucher program. The underlying premise is that by actively promoting interplay between researchers and core facilities, a dynamic feedback loop could be established that could enhance both groups, the productivity of the former and the relevance of the latter. Our primary goal was to give translational investigators what they need to pursue their immediate projects at hand.

**Methods:**

To implement this system across four noncontiguous campuses, open-source web-accessible software applications were created that were scalable and could efficiently administer investigator submissions and subsequent reviews in a multicampus fashion.

**Results:**

In the past five years, we have processed over 1400 applications submitted by over 750 individual faculty members across both clinical and nonclinical departments. In total, 1926 core requests were made in conjunction with 1467 submitted proposals. The top 10 most popular cores accounted for 50% of all requests, and the top half of the most popular cores accounted for 90% of all requests.

**Conclusion:**

Tracking investigator demand provides a unique window into what are the high- and low-priority core services that best support translational research.

## Introduction

Core facilities play crucial roles in carrying out the academic research mission by making available to researchers advanced technologies, facilities, or expertise that would be unfeasible for most investigators to obtain on their own. Ideally, such facilities would remain in constant synchrony with the needs of individual researchers. However there is no steady state in research, only one of constant flux. As such, core facilities must undergo periodic cycles of renewal in order to stay relevant. What today may be viewed as a critical technology, tomorrow may be either commercially commodified or rendered obsolete.

Recognizing the importance of core facilities, many academic institutions set aside a portion of their infrastructure budget to support them. Typically, an expert committee manages and distributes these funds. Core directors apply to this committee citing various performance metrics achieved, and support is then disbursed accordingly. Though time tested, the problem with this core-centric approach is that researchers’ voices are both attenuated and delayed. While attentive core directors are experts at applying the latest technical innovations in their fields, it is researchers themselves that can best attest to what is needed to drive their projects forward.

With this perspective in mind, the Clinical and Translational Science Institute (CTSI) at the University of California, Los Angeles (UCLA) created a Core Voucher program that would engage biomedical researchers at the outset. The intention was that investigator responses would dictate which core facilities receive CTSI funds, providing another approach to guide investments in core resources. The underlying premise of this program is that by actively promoting interplay between researchers and core facilities, a dynamic relationship could be established that could benefit both groups. Our primary goal was to ensure that translational investigators received the services they needed to address their immediate tasks at hand. If successful, we hoped that this system could also incentivize the broader UCLA CTSI research community to engage in translational research.

Herein describes our six-year experience in implementing a Core Voucher program across four noncontiguous campuses. Open-source web-accessible software applications were created and progressively refined over 17 request for application (RFA) cycles, that could efficiently administer investigator submissions and subsequent reviews. Not only has the Core Voucher program been well received by both investigators and core directors, but the resulting data provide a dynamic and unbiased view of the core services most valued by UCLA CTSI translational researchers.

## Materials and Methods

### Solicitation, Review, and Selection

#### Intercampus RFA

Each Core Voucher cycle starts with a widely broadcasted RFA to the greater UCLA CTSI research community encompassing medically-related schools, as well as physical science and engineering schools across four different campuses: Cedars-Sinai Medical Center, Charles R. Drew University, LA BioMed at Harbor-UCLA, and UCLA Westwood. Outreach includes announcing the RFA through the UCLA CTSI newsletter and website, and general email blasts through various schools and departments. All investigators engaged in translational research are encouraged to apply regardless of their departmental or school affiliations.

Investigators complete a secure online application consisting of: (i) description (2000 characters) of a translational research project that will utilize one or more of the 73 active core facilities located across the UCLA CTSI; (ii) budget justification (500 characters) estimating costs for requested core services up to a maximum total of $10,000; (iii) timeline for obtaining samples, specialized reagents, constructs, or technology required for the core services requested (500 characters); and (iv) investigator biosketches. The one or more cores needed are selected from a sorted checkbox list that reflects those listed on the UCLA CTSI website. This curated list of cores is reviewed annually to ensure those listed remain operational, and to add/remove cores as appropriate. To accommodate requests for cores not listed, “other” checkboxes with write-in functionality are also included. Each investigator is limited to submitting one proposal per RFA cycle.

Each proposal is independently evaluated by three reviewers, two from the applicant’s home campus and one from a different campus within the UCLA CTSI. Proposals are evaluated according to three criteria: (i) innovation and approach (how novel is the approach either technically or conceptually); (ii) translatability (does the proposal address a clinically relevant problem); and (iii) core utilization (is the proposed project ready to use the requested core services immediately upon award). Each criteria is given a numerical score based on a 1-to-5-point scoring scale, where 1 = exceptional. In general, the overall score is the sum of the component scores. However, to account for attributes that may fall outside of component scores, reviewers are given the option of adding or subtracting 1 point from the sum of the component scores, in calculating the overall score.

Upon completion of review, rank order lists sorted by mean overall score are generated. Because each of the participating campuses in our Core Voucher program manages their own budget, separate rank order lists are created for each campus. Each campus then sets their own pay lines and the proportion of young to senior investigators that will be awarded. Priority consideration for young investigators is handled in a campus-specific manner. At Cedars-Sinai, young investigators, defined as faculty at the Assistant Professor or equivalent level, are given a 0.5 point advantage in mean overall score, while at the other campuses, a separate rank order list is created for young investigators, faculty who are within five years of faculty appointment.

All investigators receive a report containing the award decision, their mean component and overall scores, and reviewer comments. One-on-one consultations to discuss reviewer feedback are available upon request. Core facility requests of the awardees are aggregated and core directors are sent a list of investigators who received voucher credits. As each investigator spends these credits over the 6–8-month award period, the CTSI directly reimburses the cores after requested services have been performed.

#### On-demand RFA

On-demand RFAs run for 2 months during which at any time, investigators could submit proposals using the same online submission forms as the intercampus RFAs. However, unlike the intercampus RFAs, proposals are reviewed as they are submitted in the order in which they are received. In this way, scores along with award decisions are returned to investigators within 2 weeks of proposal submission. Pay lines for each on-demand RFA are calculated based on the previous intercampus RFA that had occurred earlier in the same year.

### Program Analysis

Core Voucher awardees receive a longitudinal scientific achievement survey for outcomes (e.g. publications, follow-on funding, and patents) attributable to the UCLA CTSI support they received, up to three times over five years following the award. Since 2012, the longitudinal scientific achievement survey has been deployed four times, most recently in February 2018. Across these four surveys, 212 follow-on grants were reported by investigators who received at least one Core Voucher award between 2011 and 2016. Of these, 162 were verified by the UCLA CTSI Evaluation group from third-party sources (e.g. National Institutes of Health (NIH) RePORTER). To standardize the full amount of each reported follow-on grant, the total cost (direct and indirect costs) for all years was calculated. Of the 162 verified grants, 116 were identified as being potential candidates for attribution to a Core Voucher award, based on the timing of the Core Voucher award period and the follow-on grant start date. Attribution of a Core Voucher Award to investigator-reported follow-on funding was then validated by manually analyzing and comparing the scientific objectives of the awarded Core Voucher proposal to that of the reported follow-on grant. This process resulted in 98 instances of follow-on funding.

## Results

### A Large Number of Proposals from a Diverse Investigator Population Has Been Processed

**Fig. 1.**
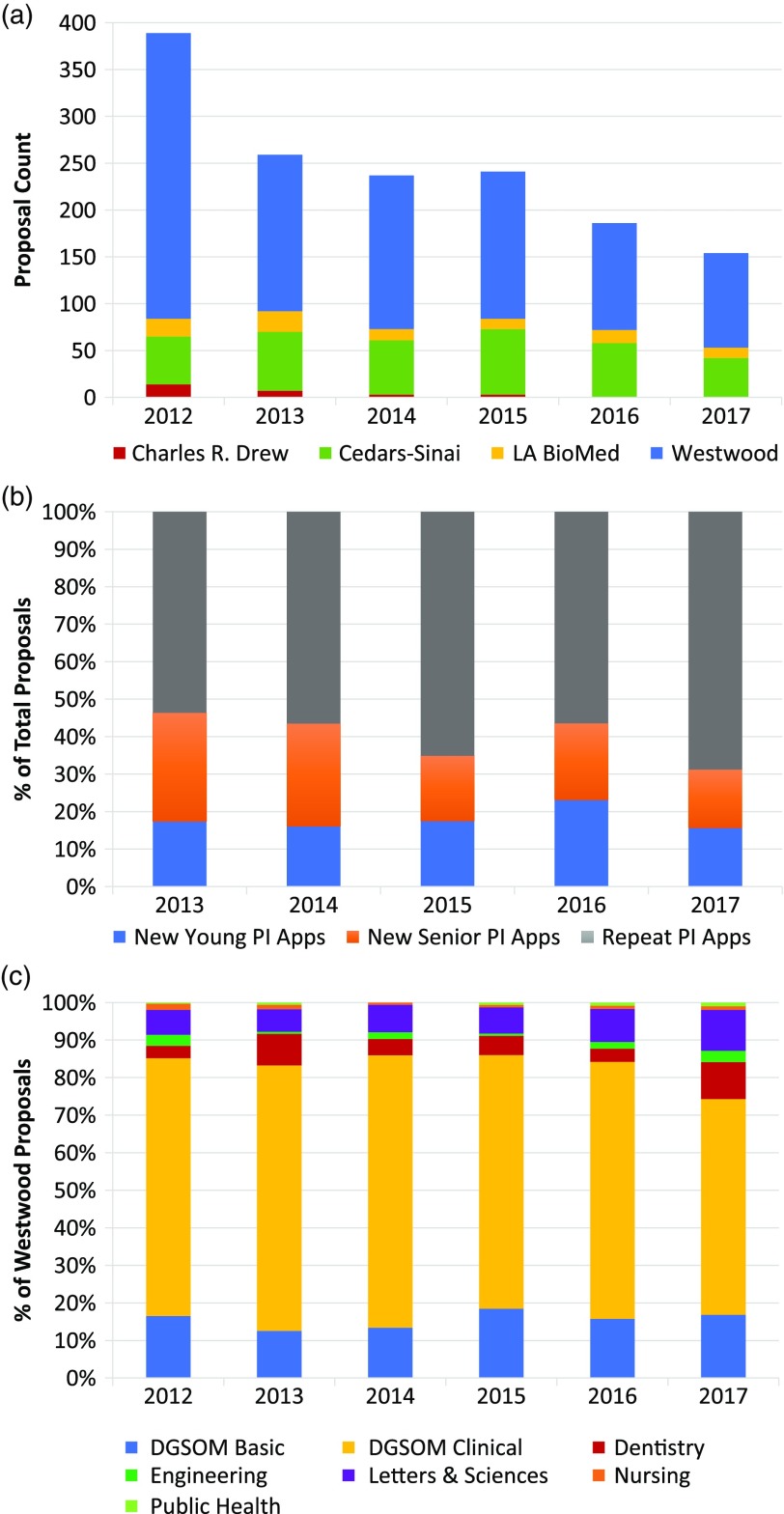
Proposal distribution and diversity over time. (a) After an initial surge at the start of the program, the number of applications from each UCLA CTSI campus has been stable over time and approximates the size of the respective investigator pools. Data represent campus-specific and intercampus requests for applications (RFAs). (b) Over the last three intercampus RFAs, the proportion of new principal investigators (PI) per cycle has remained relatively stable, and equally split between young and senior investigators. (c) At the Westwood campus, a heterogeneous mix of basic science and clinical investigators has also remained relatively stable across RFAs. Data represent Westwood-specific and intercampus RFAs.

Since its inception in 2011, the UCLA CTSI Core Voucher program has processed a total of 1545 proposals. Following an initial surge at the start of the program, the number of proposals has been relatively steady (Fig. 1(a)). The slight falloff in submissions in the last two years coincided with having only one RFA cycle per year whereas in previous years, there were multiple cycles. The relative proportion of proposals received from each campus has also been stable over time and approximates the size of the investigator pools at each location.

Since 2012, when a uniform, online application and review process was implemented across the campuses, a total of 774 individual faculty submitted proposals to the Core Voucher program: Cedars-Sinai, 168; Charles R. Drew, 15; LA BioMed, 41; Westwood, 550. Across the 13 RFA cycles since 2012, 28% of proposals were submitted by young investigators (Cedars-Sinai faculty at the Assistant Professor or equivalent level, and at the other campuses, faculty who are within five years of faculty appointment). Interestingly, the proportion of applications from faculty who had not previously applied has remained relatively stable over the last three intercampus RFAs, ranging from 33% to 41% (Fig. 1(b)). These new investigators were equally split between young and senior investigators. A total of 403 awards were made over this period, 187 to young investigators and 216 to senior investigators. Average success rates for young and senior investigators across the intercampus RFAs from 2013 to 2017 were 35% and 26%, respectively.

**Fig. 2.**
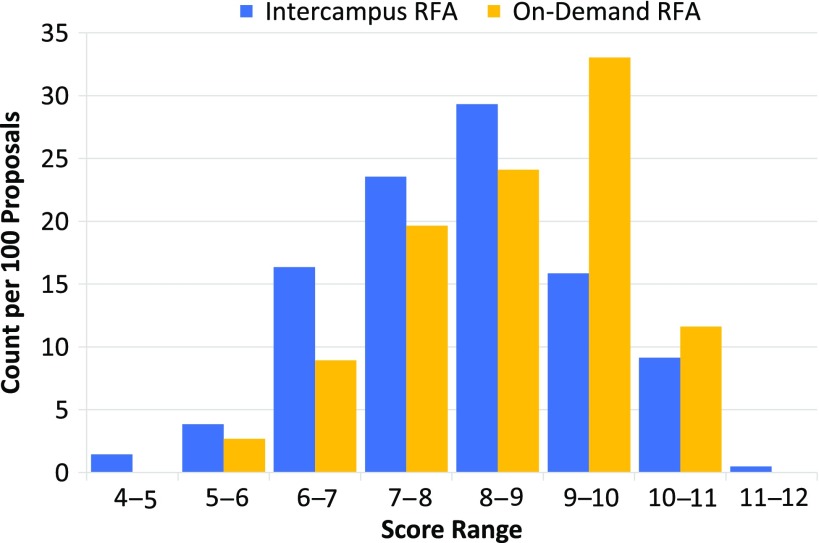
Scoring distribution between intercampus and on-demand requests for applications (RFAs). When normalized, analysis of scoring distribution between intercampus and on-demand RFAs revealed a normal distribution for intercampus RFAs, whereas on-demand RFAs showed more skewing toward higher scores.

At the Westwood campus, clinical and nonclinical departments and schools are well defined. A retrospective analysis of proposals by department/school revealed a relatively stable but heterogeneous mix of basic science and clinical investigators where the proportion of clinical to basic science proposals ranged between 2:1 and 3:1 (Fig. 1(c)). Basic science departments within the School of Medicine constituted the majority of nonclinical departments, followed by departments within the College of Letters and Sciences.

### Both Batch and On-Demand RFA Workflows Were Investigated

Our intercampus RFA is structured as a sequential batch process whereby review does not start until all proposals are received. While all efforts are made to efficiently process proposals, there still can be up to a 10-week interval from when a proposal is submitted to when reviews and scores are returned to the investigator. In response to feedback from the UCLA CTSI External Advisory Board and recognition that neither inspiration nor the pace of biomedical research conforms to time schedules, an on-demand process was piloted on the Westwood campus in 2014 and 2015 that would provide more immediate feedback.

Each of the two pilot on-demand RFAs received a similar number of proposals: 2014, 56; 2015, 57. The cohort of investigators that submitted on-demand proposals was demographically similar to those that applied through the intercampus RFAs. For example, 35% of the on-demand proposals were from young investigators as compared to 31% seen in the 2014–15 intercampus RFAs. There was a slightly higher proportion of proposals from investigators in clinical departments than that seen in intercampus RFAs (84% vs. 71%). Comparison of mean overall scores showed a difference between the on-demand and intercampus RFAs (8.4 ± 1.3 vs. 8.0 ± 1.4, respectively). Analysis of score distributions revealed a more pronounced difference between the two workflows (Fig. 2). The intercampus batch review process approximated a normal distribution whereas the on-demand scores showed more skewing toward numerically higher scores.

### Investigator Requests Dynamically Reflect Core Demand

The UCLA CTSI primarily serves as a clearing house for core services. A website is maintained with a list of descriptions and links to the approximately 70 cores that translational researchers most often seek. This list is in a constant state of renewal as cores periodically turn over. In the last 5 years, 15 cores were retired or consolidated with pre-existing cores, and 19 new cores came online (Supplementary Table 1).

**Table 1.** Core requests by year. Heat-map representing color-coded levels of differentially requested cores over five years. Cores are listed in the order of data point representation on Fig. 3(a)

Core name	Core category	2012	2013	2014	2015	2016	2017
Technology Center for Genomics & Bioinformatics	Genetics	11.1	18.5	24.5	15.4	13.4	13.0
Genomics Core	Genetics	4.1	8.5	6.8	9.1	10.8	10.4
Imaging Core	Images	3.6	7.3	6.8	8.7	6.5	8.4
Westwood_Other	Other	6.9	7.7	7.6	5.4	5.4	5.8
Flow Cytometry Core Laboratory	Cells	9.5	3.9	5.1	5.4	6.5	5.2
Neuroscience Genomics Core	Genetics	4.6	7.7	3.8	6.6	3.8	7.1
Molecular Screening Shared Resource	Molecules	8.0	3.9	2.5	4.6	6.5	7.1
Translational Pathology Core Laboratory	Humans	6.9	5.8	4.2	5.8	6.5	3.2
Advanced Light Microscopy/Spectroscopy and Macroscale Imaging Facilities	Images	5.4	2.7	5.1	2.9	5.9	9.7
DNA Microarray Core	Genetics	14.9	2.7	3.4	1.2	1.6	1.3
Flow Cytometry Core	Cells	2.1	5.0	6.3	5.8	2.7	1.9
Confocal & Two-Photon Fluorescence Microscopy Core	Images	1.8	4.6	3.0	4.1	5.4	4.5
Electron Imaging Center for Nanomachines	Images	3.1	3.5	3.8	2.1	3.8	6.5
Ahmanson Lovelace Brain Mapping Center	Images	2.3	3.5	2.5	3.3	6.5	4.5
Mass Spectrometry and Biomarker Discovery Core	Images				6.2	6.5	4.5
Preclinical Imaging Technology Center	Images	3.1	2.7	1.3	2.9	4.3	1.3
Biobank & Translational Research Core	Cells				2.9	5.4	4.5
Behavioral Testing Core	Animals	1.3	1.2	3.0	2.9	1.1	3.2
Induced Pluripotent Stem Cell Core	Cells	0.8	2.3	3.4	1.2	2.7	0.6
Immunogenetics Center	Humans	1.8	3.1	3.0	0.4	1.1	1.3
Mass Spectrometry and Proteomics Laboratory	Molecules	2.3	1.2	0.4	2.5	2.7	1.3
Electron Microscopy Services Center	Images	1.8	0.8	1.3	2.1	2.2	1.9
Mouse Physiology Laboratory	Animals	2.8	1.9	1.7	0.8	1.6	
Statistical/Biomathematical Consulting Clinic	Computations	1.8	2.3	2.1	0.8	1.1	0.6
Vector Core	Cells	1.8	1.5	2.1	0.8	0.5	1.9
Computing Technologies Research Laboratory	Computations	1.5	1.5	0.8	0.8	2.7	1.3
Cedars-Sinai_Other	Other	0.5	2.3	5.9			
Clinical and Translational Research Laboratory	Humans	1.5	4.6	1.3	1.2		
Mouse Genetics Core	Animals	1.8	2.3	1.3	2.1		0.6
Metabolomics and Proteomics Center	Molecules				0.4	1.6	4.5
Pasarow Mass Spectrometry Laboratory	Molecules	1.3			1.7	2.2	1.3
Department of Medicine Statistics Core	Computations			0.4	0.8	2.2	2.6
Microscopic Techniques Laboratory	Images	1.3		0.4	0.4	3.2	0.6
Bioimaging and Immunotherapeutics Research Core	Images	1.3	0.4	0.4	1.7	0.5	1.3
Nano and Pico Characterization Laboratory	Images	0.8		1.7	0.4	0.5	1.9
Cellular Bioenergetics Core	Cells	2.3	0.4	0.8	0.4	0.5	0.6
Semel Institute Biostatistics Core	Computations				2.9	0.5	1.3
Computed Tomographic Core	Humans		1.2	0.8	0.8	1.1	0.6
Proteome Research Center	Molecules				0.8	1.1	2.6
Guenther Molecular Biology Core	Molecules	1.0	1.2	0.4	1.2	0.5	
Center for Aids Research Humanized Mouse Core	Animals	1.5	0.4	0.8	0.4	0.5	0.6
Zebrafish Core	Animals	0.3		0.4	1.2	1.1	1.3
LA BioMed_Other	Other	0.5	0.4	1.3		1.1	0.6
Materials Characterization Laboratory	Molecules	0.3	0.4	0.4	0.4	1.1	1.3
Immuno/BioSpot Core	Cells				0.8	1.6	1.3
Informatics Center for Neurogenetics and Neurogenomics	Computations	0.8	1.2	1.3		0.5	
Peptide Synthesis Core Facility	Molecules	0.5		0.4	0.4	1.1	1.3
Pathology Research Portal	Humans			0.4	1.2	1.1	0.6
Molecular Therapeutics Core	Molecules	0.5	0.4			0.5	1.9
Magnetic Resonance Facility	Molecules		0.8	0.4	0.8	0.5	0.6
Endocrine and Metabolic Research Laboratory	Humans	0.8	0.8		0.4	1.1	
Metabolism and Mitochondrial Research Core	Cells				1.2	1.1	0.6
Translational Research Imaging Center	Images			0.4	1.2	0.5	0.6
Center for Aids Research Virology Core Laboratory	Cells	0.3		0.4	0.4	1.1	0.6
Biological Samples Processing Core	Humans	1.0	0.8	0.4	0.4		
X-ray Crystallography Core Facility	Molecules	1.3		0.4	0.8		
Macromolecular Crystallization Facility	Molecules	1.0		0.4	0.4	0.5	
Protein Expression Technology Center	Molecules		0.4	0.8	0.4	0.5	
Biobehavioral Research Core	Animals				0.4	1.1	0.6
Biostatistics & Bioinformatics Core	Computations					1.1	0.6
Tissue Culture Core	Cells	0.3	0.8	0.4			
Biomedical Mass Spectroscopy Facility	Molecules				0.8	0.5	
Microelectronics Shop	Shops	0.3		0.4		0.5	
High-Resolution Tissue Respirometry Core	Cells				0.4		0.6
Surface Plasma Resonance Core	Molecules	0.3	0.4		0.4		
Biochemistry Instrumentation Core Facility	Molecules	0.8					
Biological Chemistry Imaging Facility	Images	0.3	0.4				
Exercise Physiology Research Laboratory	Humans				0.4		
J.D. McCullough Crystallography Laboratory	Molecules						
Large Animal Core	Animals						
Center for Computer Vision and Imaging Biomarkers Laboratory	Humans						
Center for Human Nutrition	Humans						
Pulmonary Function and Cardiopulmonary Exercise Testing Core Lab	Humans						

**Fig. 3.**
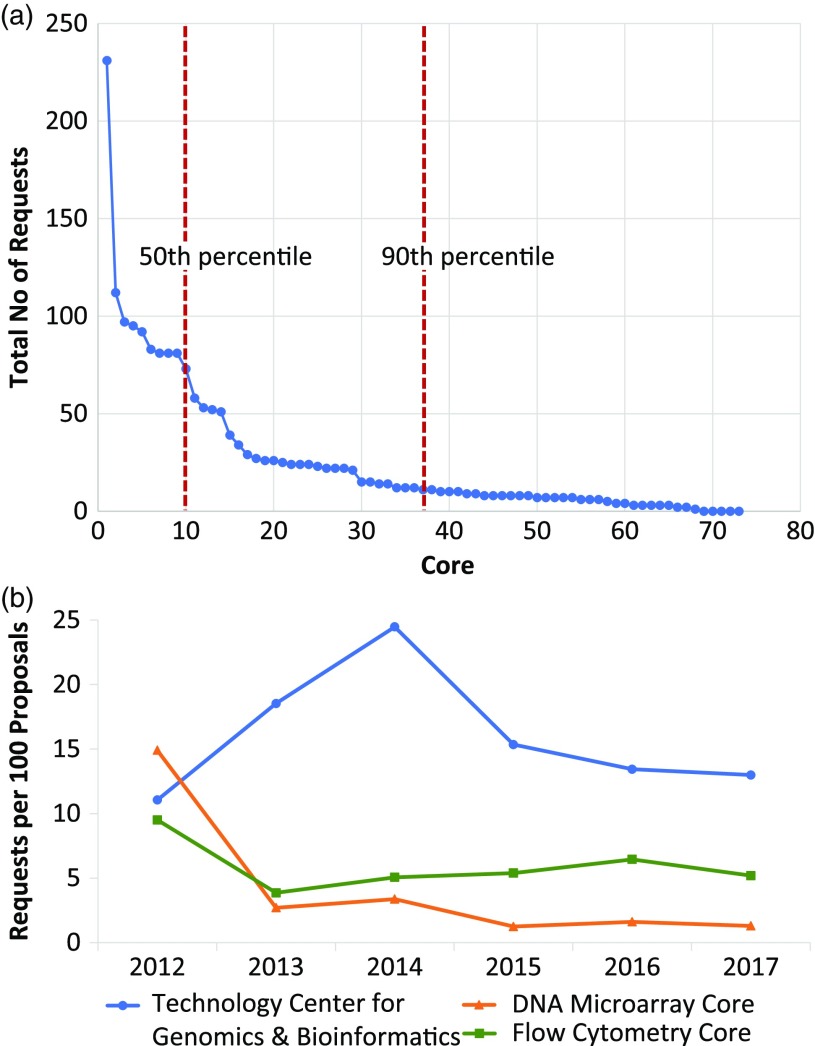
Five-year overview of core requests. (a) Graphical representation of the absolute total number of requests (*y*-axis) per individual core (*x*-axis). Each data point represents a distinct core. The top 10 most popular cores accounted for 50% of all requests. Further, the top half of the most popular cores accounted for 90% of all requests. (b) Following normalization of the number of requests for an individual core, profiles of individual core requests demonstrate a near-linear trend for three representative cores: the Technology Center for Genomics & Bioinformatics, the DNA Microarray Core, and the Flow Cytometry Core.

From 2012 through 2017, 1926 core requests were made in conjunction with the 1467 submitted proposals. Rank order aggregate analysis of these data showed that the top 10 most popular cores accounted for 50% of all requests (Fig. 3(a)). In addition, the top half of the most popular cores accounted for 90% of all requests. Further parsing the core request data by time revealed year-to-year variation (Table 1) that could be further explored at the individual core level (Fig. 3(b)). Of note, four genetics/genomics cores were contained within the top 10 cores requested.

### The Core Voucher Program Actively Promotes Translational Research

To assess our Core Voucher program’s impact on the translational science landscape, investigators are longitudinally surveyed for outcomes up to three times over five years following the award. For the period from 2011 to 2016, we expended approximately $4.6M in infrastructure and administrative costs to build and manage the program, and Core Voucher awards. Rigorous manual curation of data reported by investigators who received at least one Core Voucher award in this timeframe indicates that to date, this support was a contributing factor in obtaining $139.6M in follow-on funding, and publications in high-impact journals including *Science, New England Journal of Medicine*, and *Nature*.

The following anecdotes represent examples of impact on both young and senior investigators:

- A young investigator developing a therapeutic for Sanfilippo B Syndrome received ongoing support through the Core Voucher program, being awarded multiple Core Vouchers from 2012 through 2015. Notably, feedback from her unfunded NIH R01 grant application indicated a major weakness that could be addressed by a key experiment. She used the funds from her first Core Voucher award at a core located at a partner institution to obtain the necessary data. Upon resubmission of her NIH R01 grant, these data were noted as a key strength for the approach. The grant scored within the fifth percentile and was funded. With continued nurturing and support, she has now obtained multiple grants and published several manuscripts, and has taken a compound to clinical trials.^1^ ^–^ ^4^
- A senior, highly successful basic scientist requested a consultation to discuss her application when it was not awarded upon her first submission. Following the consultation and being steered toward a translational application for her project, she was awarded on a subsequent submission. The Core Voucher award enabled her to fund a key study, the imaging of telomerase using a special, high-powered microscopy technique called cryo-electron microscopy. This work was later published in *Science* and has the potential to inform development of anticancer and antiaging therapies.^5^ ^,^ ^6^

## Discussion

The primary focus of our Core Voucher program is to facilitate translational research through support of core services. We have taken a novel approach to this by incentivizing investigators to engage in translational research through directly linking core subsidies to investigator-defined projects. The 1926 core requests made in conjunction with the 1467 proposals submitted in the past 5 years speak to the innovation and productivity of the translational researchers and the relevance of core facilities.

Directly quantitating the depth and breadth of our research community engaged in translational research is difficult. One approach is to track the proportion of investigators that are applying to the Core Voucher system for the first time. As we reach the limit of the pool of translational investigators, we would expect this proportion to decrease. Our finding that the proportion of new investigators has remained stable over the last three intercampus RFA cycles would suggest that we have not yet fully sampled the translational research community across the UCLA CTSI campuses. Although an expanding translational research community is only one possible explanation for this observation, we are encouraged by this trend and will continue to follow this metric closely in the future.

The size of the UCLA CTSI research community and the fact that it is distributed across four physically distinct campuses drove many of the logistical decisions in implementing our Core Voucher system. Building a scalable, uniform multicampus Core Voucher submission and review process supported by web accessible software was a key component to our success. We opted for a “home-grown” open-source application that is mature and readily configurable. After all reviews had been submitted, the data were exported into a separate relational database to generate rank order lists. This hybrid approach offered not only the accessibility required for our transactional processes across both intercampus and on-demand RFAs, but also the flexibility to meet our evolving analytic needs. These software tools also had a positive impact on administrative efficiency. Even at a scale of hundreds of proposals per RFA, this does not present an undue burden to our support staff. A typical cycle requires only 0.5 of an FTE (full time equivalent) from beginning to end.

To respond quickly to investigator needs, limiting the turnaround time for the RFA process was a major focus from the outset. To accomplish this goal while anticipating a review load of 150–200 proposals per cycle, the decision was made to limit each eligible principal investigator to one application per RFA and to keep the application process as brief as possible. Over the course of the program, a coinvestigator category was added to recognize the contribution of postdoctoral fellows and other junior investigators to driving projects forward. The project description was limited to 2000 characters including white spaces, which is approximately 300 to 350 words. The brief application is in keeping with a general trend on the part of NIH and others toward shorter applications that require applicants to focus their proposals to a minimum effective size. While most investigators welcomed this approach, many found that achieving this level of brevity, while maintaining effectiveness, was challenging. The most frequent challenge encountered was the ability to articulate the translational relevance as well as the innovation and approach of the project. Many investigators welcomed the opportunity for one-on-one consultations to discuss such deficiencies, which resulted in improved application outcomes in subsequent cycles.

Even with a streamlined process, it still takes approximately 6 weeks from the time an intercampus RFA ends, to when award notifications are distributed. We reduced this interval to 2 weeks using an on-demand RFA process but this change in procedure had consequences in the score distribution. It is possible that this was due to a change in reviewer selection but the high level of overlap between these two processes makes it unlikely. The proposals from Westwood during the intercampus RFAs were reviewed by two of the three possible Westwood reviewers while the on-demand proposals were scored by all three Westwood reviewers. The skewed score distribution seen with the on-demand RFAs may reflect a change of how the same reviewers score proposals. When tasked with evaluating a batch of 30–100 proposals as with the intercampus RFAs, reviewers have the ability to cross compare immediately at hand. However for the on-demand RFAs they had to evaluate 5–7 proposals in relative isolation each week. This increased level of difficulty may have prompted a tendency toward more conservative scoring. Given our perceived limited benefits of the on-demand RFA, we have now opted to focus our efforts on outreach for the intercampus RFA.

Supporting core services that best support translational research is the central rationale for developing our Core Voucher program. In an effort to boost the overall quality of translational research across our consortium, a memorandum of understanding (MOU) to standardize core pricing for UCLA CTSI investigators across our sites was implemented. The MOU not only enables greater access to core resources and expertise, but also provides an opportunity for cross-campus collaborations.^7^ Approximately 10% of intercampus RFA applications requests services for a core located at a partner institution.

Analyzing the 1926 core requests made by investigators in the context of actual research projects submitted from 2012 to 2017 has provided unique insight into which services were considered to be high or low priority for translational research. The finding that the top 10 most requested cores account for half of all requests was surprising. The observation that all four of the genetics/genomics cores were contained in this top 10 was not, and reinforces the profound impact that high throughput nucleotide sequencing technologies continue to have in translational biomedical research.

Core services lie at a metastable interface between what is needed now by biomedical investigators and what can be commodified by industry. They are in a constant squeeze between having to offer technologies that are still relevant, while at the same time remaining price/performance competitive. For example, the cores offering high throughput nucleotide sequencing are currently doing a robust business in meeting a deep demand. However if this technology can be scaled to an industrial level at which smaller university-based cores cannot compete, the picture could change dramatically and in a short period of time.

Tracking investigator demand is a sensitive and direct way of assessing ongoing core health. Even for cores that independently monitor their own usage statistics, data from the Core Voucher program provide a more broad-based gauge of demand since it does not rely on investigators having funding in hand. In addition, because our tabulated core requests are tied to specific projects, we gain insight into what core services are needed for translational research. In this regard, the absolute number of requests is only one metric of performance. Demonstration of a consistent demand over time is also another indicator that the services provided by a core continue to be needed. This information in addition to their own tracking data will be valuable to core directors as they continue to undergo cycles of reinvention and renewal in order to stay afloat. At the institutional level, aggregate views of these data that comparatively track all core services will likely be most useful. Supporting a mixed portfolio of high-, medium-, and even low-volume core services with stable demand profiles over time will provide sufficient diversity and coverage that will best meet the needs of investigators engaged in translational research.

One approach to measuring a program’s impact is through assessing follow-on funding. Verification of investigator-reported follow-on funding via survey was followed by expert manual curation as the population of candidates was progressively winnowed down to identify voucher award/follow-on award pairs with a high degree of relatedness. Even with this rigorous approach, we recognize the limitation of such analyses. At this point, the most we can infer is correlation but not causation since performing the counterfactual experiment is not readily done. Had these investigators not received their Core Voucher awards, whether or not they would have been successful in obtaining follow-on awards is difficult to predict with certainty. Nonetheless, our follow-on funding analysis as well as individual case stories attest to an ongoing positive impact in promoting translational research at both programmatic and investigator levels. Perhaps the most striking indication of the UCLA CTSI’s Core Voucher program success lies in the fact that for the last two years, it has been entirely institutionally supported. Faced with the inability to continue supporting the program with grant funds upon our CTSA renewal due to programmatic restrictions, all actively participating campuses within our consortium secured local funds to continue the program.

## Supplementary material

For supplementary material accompanying this paper visit https://doi.org/10.1017/cts.2019.4. click here to view supplementary material
