# Describing the dynamic translational science landscape through core voucher utilization — ERRATUM

**DOI:** 10.1017/cts.2019.420

**Published:** 2019-11-27

**Authors:** Elvira L. Liclican, Scott G. Filler, Jonathan Kaye, Christopher T. Denny

The above article by Liclican et al. [1] published with the incorrect title for Table 1. The correct title for Table 1 is as follows:

“**Table 1.** *Core requests by year. Table displaying levels of differentially requested cores over five years (normalized per 100 application submissions). Cores are listed in the order of data point representation on Fig. 3(a)*.”

The publisher apologizes for this error.
