# Supplementary material for: Describing the dynamic translational science landscape through Core Voucher utilization
Source: J Clin Transl Sci. 2019 Jun 14;3(2-3):105–12. doi: 10.1017/cts.2019.4 (PMC6802412; doi:10.1017/cts.2019.4)
Supplement: Supplementary file 1 [file S2059866119000049sup001.pdf]

Supplementary Table 1: List of cores added to, or retired from, UCLA CTSI website. Since 2012, nineteen cores have been added to the UCLA CTSI website, while 15 have been retired or consolidated with pre-existing cores

Core Status	Core Name	Core Category
New	Biobehavioral Research Core	Animals
New	Large Animal Core	Animals
New	Biobank & Translational Research Core	Cells
New	High-Resolution Tissue Respirometry Core	Cells
New	Immuno/BioSpot Core	Cells
New	Metabolism and Mitochondrial Research Core	Cells
New	Biostatistics & Bioinformatics Core	Computations
New	Department of Medicine Statistics Core	Computations
New	Semel Institute Biostatistics Core	Computations
New	Center for Computer Vision and Imaging Biomarkers Laboratory	Humans
New	Center for Human Nutrition	Humans
New	Exercise Physiology Research Laboratory	Humans
New	Pathology Research Portal	Humans
New	Pulmonary Function and Cardiopulmonary Exercise Testing Core Lab	Humans
New	Mass Spectrometry and Biomarker Discovery Core	Images
New	Translational Research Imaging Center	Images
New	Biomedical Mass Spectroscopy Facility	Molecules
New	Metabolomics and Proteomics Center	Molecules
New	Proteome Research Center	Molecules
Retired	Mouse Pathology Core	Animals
Retired	Preclinical Biobehavioral Core	Animals
Retired	Embryonic Stem Cell / Transgenic Mice Shared Resource	Cells
Retired	Immunotherapeutics Research Core	Cells
Retired	Viral Vector Core	Cells
Retired	Genotyping and Sequencing Core	Genetics
Retired	High-throughput Genotyping Laboratory	Genetics
Retired	Carol Moss Spivak Advanced Light Microscopy Core	Images
Retired	Macro-Scale Imaging Lab	Images
Retired	Two Photon Imaging Core	Images
Retired	Functional Proteomics Laboratory	Molecules
Retired	High-Throughput Clinical Proteomics Core	Molecules
Retired	Medical Mass Spectroscopy Facility	Molecules
Retired	W. M. Keck Proteomics Center	Molecules
Retired	Glass Shop	Shops
Retired	Research Equipment and Chemical Store	Shops
